# 

**Published:** 1863-01

**Authors:** 


### CONTENTS.

### ORIGINAL CONTRIBUTIONS.

ART. I. ON OPERATIONS FOR INJURIES OF THE HEAD. By A. FISHER, M.D., of Chicago, Ill., ........................................ 1

ART. II. COMPLETE RECORD OF THE SURGERY OF THE BATTLES FOUGHT NEAR VICKSBURG, DEC. 27, 28, 29, and 30, 1862. By E. ANDREWS, late Surgeon of 1st Reg. Ill. Light Artillery, and Professor of Surgery in the Medical Department of Lind University, .................... 12

### EDITORIAL.

American Medical Association, .................................... 58
Illinois State Medical Society, .................................... 59
Circular from Henry Wing, M.D., Sec'y of Board of Medical Examiners, 60
New Books, .................................... 60
Communications, .................................... 60
Artificial Limbs, .................................... 61
Medical Qualifications, .................................... 61
Circular to Physicians, .................................... 61
Use of Tobacco, .................................... 63
Reported Killed, .................................... 64

